# Clinical value of ultrasound for the evaluation of local recurrence of primary bone tumors

**DOI:** 10.3389/fonc.2022.902317

**Published:** 2022-09-15

**Authors:** Yu Wang, Ping Yu, Feifei Liu, Yuqin Wang, Jiaan Zhu

**Affiliations:** Department of Ultrasound, Peking University People’s Hospital, Beijing, China

**Keywords:** ultrasound, local recurrence, primary bone tumors, metal implants, limb salvage

## Abstract

**Background:**

Early detection of local recurrence would improve the survival rate of patients with recurrent bone tumors. There is still no consensus on how to follow up after surgery of primary malignant bone tumors. Therefore, the purpose of this study is to evaluate the diagnostic value of ultrasound (US) for local recurrence after limb salvage by comparing it with other imaging modalities.

**Methods:**

We retrospectively reviewed the medical records of patients who were regularly examined by US in our hospital after primary bone tumor surgery from January 2016 to December 2019, some of which underwent x-ray, computed tomography (CT), or ^99^mTc-MDP bone scan. Recurrence was determined by pathologic confirmation. The cases were considered a true negative for no recurrence if no clinical or pathologic evidence for recurrence was found at least 6 months after the US examination. The Chi-square test or Fisher exact test was used to compare categorical data. *p*-values < 0.0083 were considered statistically significant.

**Results:**

A total of 288 cases were finally enrolled in our research, including 66 cases with pathologic results. The sensitivity of US was 95.0%, higher than that of x-ray (29.6%) (*p* = 0.000). The accuracy of US was 96.9%, higher than that of x-ray (85.6%) (*p* = 0.000).

**Conclusion:**

As a nonradiative and cost-effective examination, US may be used as a routine imaging method for postoperative surveillance of primary bone tumors, especially those with metal implants, if more multicenter prospective studies can be done in the future.

## Introduction

Primary malignant bone tumors, including different subtypes such as osteosarcoma (OS), Ewing sarcoma (ES), and chondrosarcoma (CS), belong to the broader category of cancers known as sarcomas (1). At present, limb-salvage surgery with tumor resection and reconstruction has become the standard treatment for high-grade extremity tumor (2–4). The purposes of limb‐salvage surgery are to reduce local recurrence and to retain good limb function, with 90% of patients undergoing limb‐salvage surgery having a success rate of 60%–80% (5). After limb salvage, the local recurrence rate was about 5.4%–13.5% (6–9). The reasons of local recurrence can be poor chemotherapy response or failure to achieve adequate surgical margins and poor response to chemotherapy (6, 8, 10, 11). It was reported that large tumor size of local recurrence was a poor prognostic factor in patients (12, 13). The prognosis of patients with local recurrence is poorer than that of patients with metastasis alone (14). Theoretically, early detection of local recurrence would improve the survival rate of patients with primary bone tumors (15).

It is hard to find the local recurrence at the reconstructed site of extremity tumor by physical examination, and therefore, it is necessary to follow up with an imaging modality (16). Although different imaging modalities have been used for the early detection of local recurrence of bone tumors, it is still difficult to distinguish post-therapeutic changes and local recurrence (17, 18). There is still no consensus on how to follow up after surgery. The ESMO Guidelines Committee’s 2018 Clinical Practice Guidelines for diagnosis, treatment, and follow-up of bone sarcomas did not set strict rules for postoperative follow-up of bone tumors, due to the different opinions in this panel of experts and the absence of any formal prospective studies (19). To the best of our knowledge, there have been few reports on the clinical significance of ultrasound (US) imaging compared with other imaging tools for detection of local recurrence in primary bone tumors. Therefore, the aim of this study is to evaluate the diagnostic value of US for local recurrence after limb salvage by comparing it with other imaging modalities.

## Methods

### Data source and patient selection

Retrospective analysis was performed on the data of patients who received US surveillance in our hospital after primary bone tumor surgery from January 2016 to December 2019. The cases were included if no clinical evidence of recurrence was found at least 6 months after the examination or there was a pathologic result. The cases were excluded if they were suspected recurrence but without pathology or lost to follow-up. Six patients were excluded, which were suspected recurrence but without pathology or lost to follow-up. A total of 272 patients (160 male and 112 female patients; median age, 21.6 years; range, 4–76 years) were finally enrolled in our research, including 226 OS, 15 CS, 25 ES, 5 giant cell tumor, and 1 sacrum tumor. The cases were considered a true negative for no recurrence if no clinical evidence of recurrence was found at least 6 months after the examination, including 222 patients. Of the remaining 50 patients with pathologic results, 2 patients were suspected of relapsing three times and 12 patients were suspected of relapsing two times. Each recurrence with a pathologic result was considered as an independent case. If one patient relapsed twice and had two pathologic results, it would be defined as two cases. If one patient relapsed three times and had three pathologic results, it would be defined as three cases. Thus, a total of 66 cases with pathologic results were finally included in the analyses. Finally, a total of 288 cases were included in the final analyses, including 66 cases with pathologic results and 222 cases with no evidence of recurrence after 6 months of follow-up. The recurrence time was calculated from the last US scan to the previous surgical or puncture pathological results.

### Diagnostic criteria of different imaging methods

All the images were reviewed by two skilled radiologists (Yu Wang and Ping Yu) blinded to the pathological information. Each reader independently analyzed the images first, and then reviewed the cases with discrepancy in their initial evaluation together. Finally, a consensus was reached after discussion.

Comparison with previous images is crucial for differentiation between postoperative alterations and subtle signs of recurrence. For CT and x-ray, new abnormal bone and soft tissue density compared with the previous examination is crucial for diagnosis. For US, the new mass in the deep fascial layer or destruction to the surface of bone compared with the previous examination is crucial for diagnosis. For ^99^mTc-MDP bone scan, an increased radiotracer accumulation compared with the previous examination is crucial for diagnosis.

### Diagnostic value of different imaging methods

US, x-ray, CT, and ^99^mTc-MDP bone scan were used to detect local tumor recurrence after primary bone tumor surgery. Comparison with prior imaging is crucial for differentiation between postoperative alterations and subtle signs of recurrence. If the first examination was not sure whether there was a recurrence, the results of multiple follow-ups will be used as the final diagnosis of recurrence or not. The sensitivity, specificity, and diagnostic accuracy of different imaging tools were compared. The sensitivity of an imaging is its ability to identify the cases with local recurrence correctly. The specificity of an imaging is its ability to identify the cases without local recurrence correctly. The accuracy of an imaging is its ability to differentiate the cases with local recurrence and without local recurrence correctly.

To determine the sensitivity, specificity, and diagnostic accuracy of different imaging tools, the following definitions were used:

1. True positive (TP): There was local recurrence confirmed by pathology.
2. False positive (FP): Local recurrence was suspected by imaging but not confirmed by pathology.
3. True negative (TN): There was no local recurrence confirmed by pathology or if no clinical evidence of recurrence was found at least 6 months after the examination.
4. False negative (FN): Local recurrence was not suspected by imaging but confirmed by pathology.

### The ultrasound characteristics

All sonographic examinations were performed with color Doppler US diagnostic equipment such as the Canon Aplio i800 with the i8CX1 and i18LX5 probes, the GE LOGIQ E9 with the C1-5 and ML6-15 probes, the Philips EPIQ 5 with the C5-1 and L12-3 probes, the Siemens Acuson S3000 with the 6C1 and 9L4 probes, the SuperSonic Imagine Aixplorer with the XC6-1 and SL10-2 probes, and the Toshiba Aplio 500 with the 6C1 and 12L5 probes. The linear array transducers were used for superficial tissue scanning, and the abdominal convex probes were used for deep tissue scanning. The following sonographic features were recorded for each case: the number of tumors (single, multiple), maximum tumor diameter, margin (regular, irregular), border (clear, obscure), cystic changes (absent, present), calcifications (absent, present), and bone destruction (absent, present). In some patients, US was followed up several times in a short period, and the sonographic features referred to the images of the last US imaging. The grade of blood on color Doppler was divided into four grades in the tumor: Grade 0 meant no blood flow signal; Grade 1 meant a small amount of blood flow, with one to two punctured or rod-shaped blood flow signals; Grade 2 meant that the blood flow was moderate, with three to four punctured blood flow signals or a longer vessel with its length close to or more than the tumor radius; Grade 3 meant rich blood flow, with more than four punctured blood flow signals or two longer vessels. Moreover, we also recorded the distribution patterns of tumor vascularity on color Doppler (predominantly intratumor vascularity, predominantly peritumor vascularity).

### Statistical analyses

Quantitative data reported in this work are expressed as mean, standard deviation (SD), range, or median for each value. The Chi-square test or Fisher exact test was used to compare categorical data. Bonferroni correction was used for adjustment after multiple comparisons. Since four image sets were compared in this study, *p*-values < 0.0083 (derived from 0.05 divided by 6) were considered statistically significant.

## Results

Finally, a total of 288 cases were included in the final analyses, including 66 cases with pathologic results and 222 cases with no evidence of recurrence after 6 months of follow-up. Before each pathologic examination, US was performed at all 288 (100%) of these cases at least once, x-ray at 271 (94.1%), CT at 87 (30.2%), and ^99^mTc-MDP bone scan at 217 (75.3%). CT was performed without contrast material in 29 cases (33.3%), and CT was performed with contrast material in 58 cases (66.7%). There were 60 cases of confirmed tumor recurrence by pathology, and US confirmed 57 cases. The median recurrence time proposed by US was 51 weeks.

### Comparison of different imaging methods in the detection of local recurrence

The diagnostic value of different imaging methods in local recurrence after limb salvage, especially with metal implants in primary bone tumors, is shown in **Table 1**. The specificity differences between US and x-ray/CT/^99^mTc-MDP bone scan were not statistically significant. The sensitivity differences between US and x-ray (*p* = 0.000), between x-ray and ^99^mTc-MDP bone scan (*p* = 0.001), and between x-ray and CT (*p* = 0.000) were statistically significant. The sensitivity of US was 95.0%, higher than that of x-ray (29.6%). The accuracy differences between US and x-ray (*p* = 0.000) and between x-ray and ^99^mTc-MDP bone scan (*p* = 0.000) were statistically significant. The accuracy of US was 96.9%, higher than that of x-ray (85.6%).

**Table 1:** Diagnostic value of different imaging methods in local recurrence after limb salvage, especially with metal implants in primary bone tumors.

Variables	Sensitivity (%)	Specificity (%)	Accuracy (%)
US	95.0#	97.4	96.9#
CT	92.9#	97.8	95.4
X-ray	29.6*^	99.5	85.6*
^99^mTc-MDP bone scan	72.7#	98.5	95.9#

US, ultrasound; CT, computed tomography.

*p < 0.0083, compared with US; ^p < 0.0083, compared with CT; #p < 0.0083, compared with x-ray.

### The ultrasound characteristics

**Figure 1:**
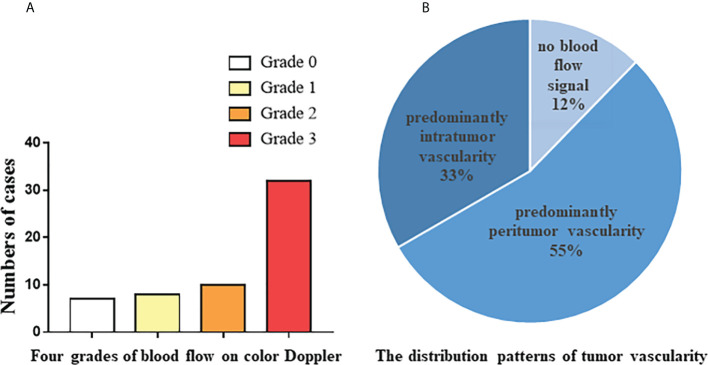
Color Doppler feutures of 57 tumor recurrence cases confirmed by pathology. **(A)** The number of cases from four grades of blood flow on color Doppler. **(B)** The distribution patterns of tumor vascularity on color Doppler.

There were 60 cases of confirmed tumor recurrence by pathology, and US confirmed 57 cases. The following sonographic features were recorded and analyzed. As shown in **Table 2**, 57.9% of tumors were single; maximum tumor diameters were 5.5 ± 3.1 cm, 24.6% of tumor margins were regular, 68.4% of tumor borders were clear, cystic changes were present in 19.3% of tumors, calcifications were present in 45.6% of tumors, and bone destruction was present in 14.0% of tumors. As shown in **Figure 1A**, Grade 4 blood flow occurred most frequently in tumors. It was remarkable that not all tumors had abundant blood flow signals, and 12.3% of the tumors had no blood flow signal. Furthermore, as shown in **Figure 1B**, according to the recorded distribution patterns of tumor vascularity on color Doppler, predominantly peritumor vascularity occurred more than predominantly intratumor vascularity.

**Table 2:** Sonographic features of 57 tumor recurrence cases confirmed by pathology.

Sonographic features	Single tumor	Maximum tumor diameter (cm)	Regular margin	Clear border	Cystic changes	Calcifications	Bone destruction
Numbers (%)	33 (57.9%)	5.5 ± 3.1	14 (24.6%)	39 (68.4%)	11 (19.3%)	26 (45.6%)	8 (14.0%)

## Discussion

Early detection of local recurrence would improve the survival rate of patients with recurrent bone tumors. It is hard to find the local recurrence at the reconstructed site of extremity tumor by physical examination, and therefore it is necessary to follow up with imaging modality (16). Although different imaging modalities have been used for the early detection of local recurrence of primary malignant bone tumors, there is still no consensus on how to follow up after surgery (17, 18). Our study showed that US surveillance was a good imaging modality for detection of local recurrence in primary bone tumors, which was better than x-ray and comparable to CT and ^99^mTc-MDP bone scan.

**Figure 2:**
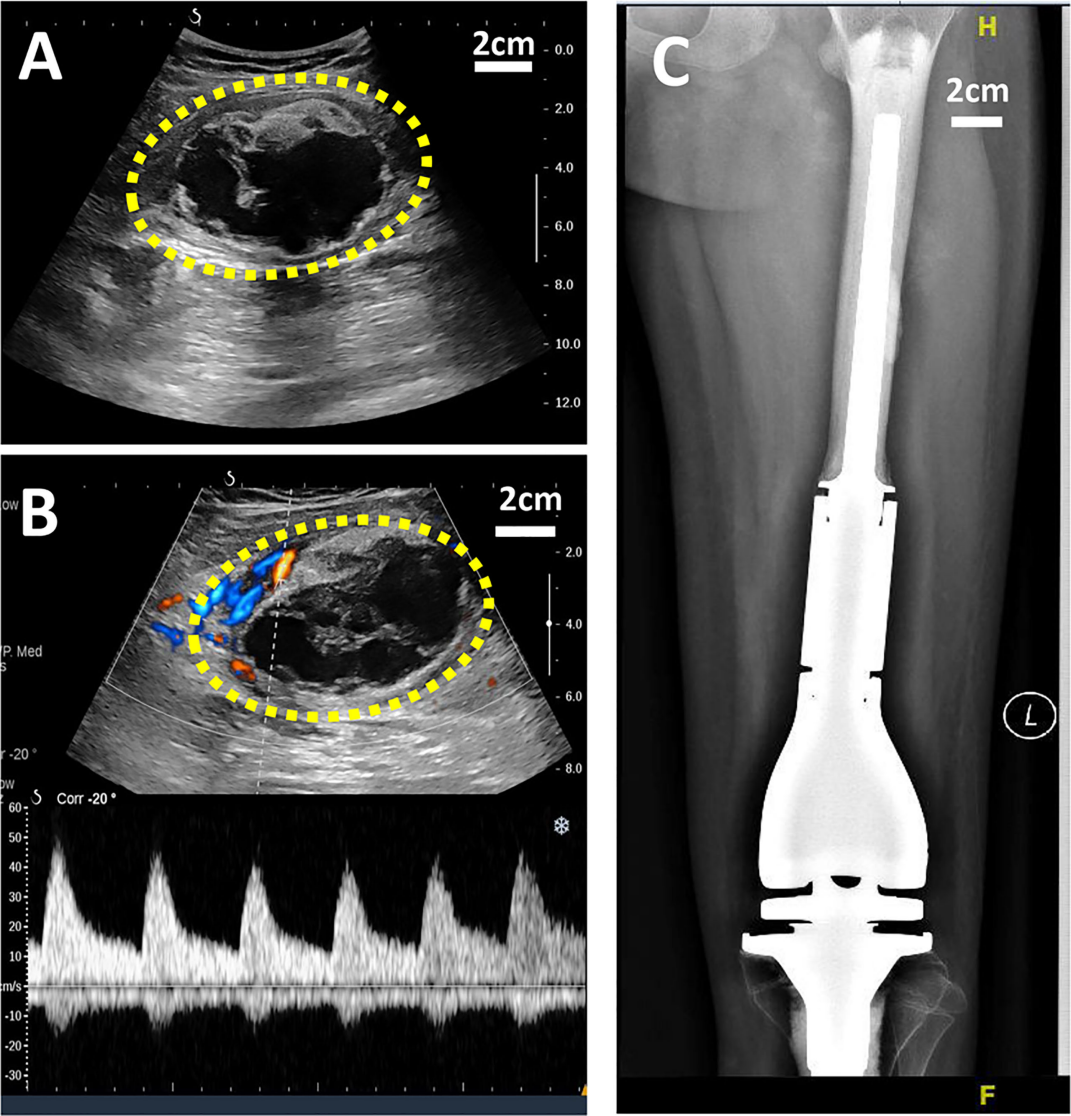
US and X-ray images of a 11-year-old girl with OS local recurrence after limb salvage with metal implants. It was easy to see the lesion in US but not in X-ray image. **(A)** A heterogeneous echogenic mass in the longitudinal plan of US image. **(B)** Color Doppler and power Doppler US image of the mass. **(C)** No obvious lesion was found I the X-ray image.

**Figure 3:**
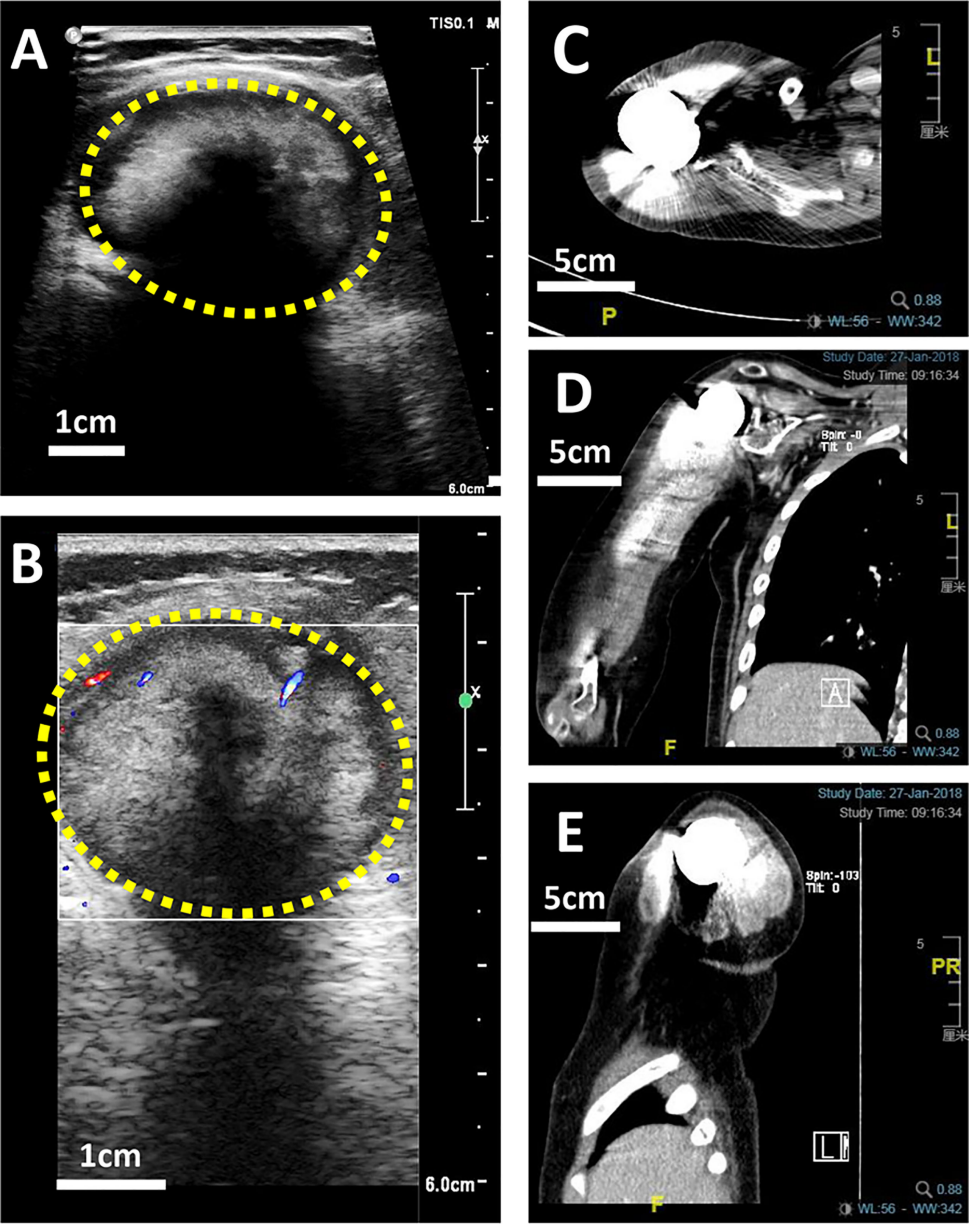
US and CT images of a 10-year-old boy with OS local recurrence after limb salvage with metal implants. It was easy to see the lesion in US but not in CT due to the imaging artifacts of metallic prosthesis. **(A)** A heterogeneous intramuscular hypoechoic mass in the right shoulder in US image. **(B)** Color Doppler US image of the mass. **(C)** The transverse plan of CT image. **(D)** The coronal plan of CT image. **(E)** The Longitudinal plan of CT image.

X-ray is a routine means of postoperative monitoring, which has advantages in showing abnormal changes in bone. Identifying a well-defined border is crucial for differentiating heterotopic ossification from local recurrence (20). However, it often misses minor lesions, bone marrow disease, and joint and soft tissue disease. As shown in **Figure 2**, it was easy to see the lesion in US but not in an x-ray image. CT has a higher spatial resolution than x-ray and can provide more information about soft tissue lesions. However, whether the benefits of using CT for early detection of lesions outweigh the risks of radiation exposure and false positives remains to be further investigated. For the cases with artificial prosthesis replacement or metal internal fixation implantation, significant artifacts are generated on CT, which affect the observation of local tissues (21, 22). As shown in **Figure 3**, it was easy to see the lesion in US but not in CT due to the imaging artifacts of metallic prosthesis. Systemic bone scanning with ^99^mTc-MDP can reveal local recurrence, systemic bone metastasis, and skipping lesions. However, the high uptake area cannot distinguish between tissue repairing response, residual disease, or recurrent lesions. A local recurrence may be found at the primary site, the stump, the resection site, or near the prosthesis. When the recurrent tumor is localized in soft tissue and does not contain sufficient mineralized osteoid, it may not be detected by conventional radiographs or even bone scans (23).

As is known to all, the real-time ultrasonography is not very useful in detecting primary bone tumors. However, US is helpful at recognizing local tumor recurrence, due to its excellent temporal and spatial resolution for soft tissue (24). Cost-effectiveness and no radiation are also very important for patients who need long-term follow-up. US used for musculoskeletal system not only can identify cystic or solid lesion, but also can evaluate inflammatory or neoplastic lesions by short-term follow-up of changes in mass size. With the application of color Doppler, US can further evaluate the blood flow inside the tumor and measure the relevant hemodynamic parameters, which is helpful to determine the pathway for needle biopsy. The observation of postoperative region by US is not limited to bone surface and surrounding soft tissue structure. US can also clearly show peripheral nerves, blood vessels, and lymph nodes, and observe whether there is traumatic neuroma, venous thrombosis, tumor embolus, regional lymph node metastasis, and so on. Another advantage of US is that it can scan the local soft tissue in all directions, show the shape of bone, and not be affected by the metal artifact of prosthesis. Theoretically, early detection of local recurrence would improve the survival of patients with recurrent bone tumors (15); postoperative imaging follow-up is an important part of bone tumor monitoring. Hence, as a cost-effective and radiation-free examination, US may be considered as a first-line imaging modality in the surveillance for patients of primary bone tumors after surgery.

Certainly, there are still several limitations in our study. First, US is the most operator-dependent modality, and it is complex to be objective on the evaluation of the findings of the single images. Second, this was a single-center study, and the sample size was not large enough. Third, data from positron emission tomography/computed tomography (PET/CT) and magnetic resonance imaging (MRI) were collected but not included in this paper, because the sample size was small. Fourth, contrast-enhanced ultrasound (CEUS) was used to determine the extent of the lesion; whether CEUS can distinguish benign masses from recurrent lesions needs further study.

In conclusion, this study showed that US is a good imaging tool for detection of local recurrence in primary bone tumors, not only because it is nonradiative and cost-effective, but also because it clearly shows local soft tissue and is not affected by metal artifacts. US can provide a reliable basis for timely adjustment of clinical treatment plan, thus improving the long-term prognosis and quality of life of patients. For postoperative surveillance of primary bone tumors, especially those with metal implants, US may be used as a routine imaging method, if more multicenter prospective studies can be done in the future.

## Data availability statement

The original contributions presented in the study are included in the article/supplementary material. Further inquiries can be directed to the corresponding author.

## Ethics statement

This study was reviewed and approved by the Ethics Committee of Peking University People’s Hospital. Written informed consent from the participants’ legal guardian/next of kin was not required to participate in this study in accordance with the national legislation and the institutional requirements.

## Author contributions

JZ participated in conceiving and designing the study. PY collected the data, YW did the analyses and drafted the manuscript. PY and YW reviewed all the images. FL and YQW handled the figures and article format. All authors contributed to the article and approved the submitted version.

## Funding

The study was supported by the National Natural Science Foundation of China (No. 82071930).

## Conflict of interest

The authors declare that the research was conducted in the absence of any commercial or financial relationships that could be construed as a potential conflict of interest.

## Publisher’s note

All claims expressed in this article are solely those of the authors and do not necessarily represent those of their affiliated organizations, or those of the publisher, the editors and the reviewers. Any product that may be evaluated in this article, or claim that may be made by its manufacturer, is not guaranteed or endorsed by the publisher.
